# The History of the Precordial Early Repolarization and Sudden Death Syndrome, Lately Named Brugada Syndrome

**DOI:** 10.3390/jcm15103903

**Published:** 2026-05-19

**Authors:** Bortolo Martini

**Affiliations:** Cardiovascular Unit, Alto Vicentino Hospital, via Garziere 42, 36014 Santorso, Italy; bortolo.martini@gmail.com

**Keywords:** Brugada syndrome, precordial early repolarization syndrome, sudden death

## Abstract

This paper intends to go through the medical history of a new syndrome, beginning from its incidental observation to the nowadays ongoing reports quickly approaching 7000 published papers. This large number makes it difficult for the researcher to correctly quote the previous significant published data, and the usual strategy is to copy and paste the last articles references. This review is mainly detailed historical research of the step-by-step journey mainly of the first three decades, with less attention to the ongoing and late scientific controversies that are indeed quoted. The new syndrome was early named “precordial early repolarization (PER) syndrome” but became popular after being renamed “Brugada syndrome” (BS). Nowadays it is classified as one of the “J wave syndromes” (JWSs). The main characteristic of this new entity was an unusual astonishing precordial coved ST segment elevation that gave rise since its first descriptions to two different pathophysiological theories, one organic and the second functional. The first theory ascribed the ST elevation to an unusual pattern of depolarization at the right ventricular outflow tract (RVOT), while the second favored an abnormal dynamic repolarization pattern. Both phenomena were sometimes linked to an ion channel genetic abnormality. In the following decades, many eminent scientists and also some excellent humble cardiologists made significant observations regarding epidemiology, laboratory, diagnostic techniques, genetic, clinical findings, histology, embryology, therapeutic approaches, and risk stratification. This rush “to be the first who” has created more confusion than certainty, and only in this last decade a more scientific and less emotional approach has led to a common acceptance of an underlying organic background that causes a strange conduction delay mainly at the epicardial level of the RVOT. “Next generation” cardiologists are in charge of further elucidating the genetic, the structural, and electrical pathophysiology, and the correct risk stratification needed to correctly identify the true patients who need a therapy and avoid unusual and dangerous treatments to healthy people with a benign strange ECG.

## 1. Introduction

**Figure 1:**
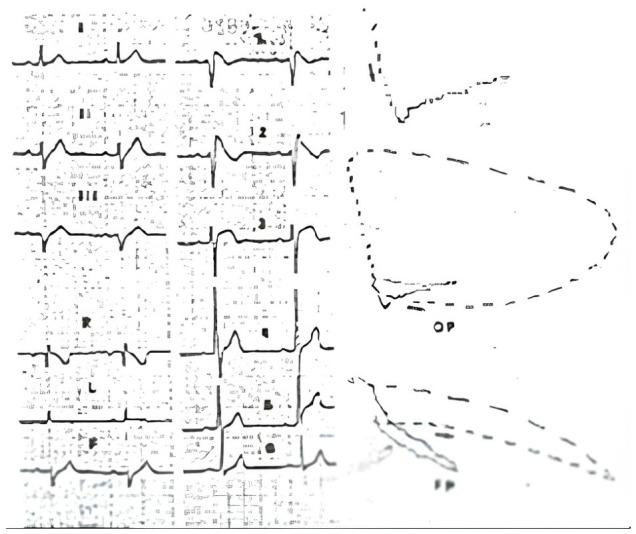
The ECG and Vectorcardiogram (VCG) of the first patient with the syndrome of PERS (3). The ECG shows a coved precordial ST elevation, absent s wave in V6, left axis deviation. PR 200 msec. The VCG shows a right-posterior-superior delay of the terminal QRS [3].

A cardiac syndrome is not an isolated electrocardiographic (ECG) pattern but the association between symptoms and signs as taught by the Persian philosopher Avicenna (980-1037) in his masterpiece “The Canon of Medicine”. Since 1988, a new syndrome was firstly presented in Italy, Japan, and Spain, reporting cases of aborted sudden death with a strange ECG: a coved ST elevation of the precordial leads, previously described as benign PERS [1,2,3,4,5,6,7,8]. The spontaneous typical ECG pattern [3] lately named type 1 (Figure 1), is not the most frequent “typical” ECGpattern. In manypatients and in asymptomatic people affected by the syndrome the ECG-pattern is often a type 2,3 (saddle-like ST elevation) and drug-induced type 1.

The main characteristics of the new syndrome included (1) middle-aged men with familial occurrence; (2) Ventricular Fibrillation (VF) at rest, or syncope of unknown origin. A major effort must be made to differentiate arrhythmic syncope from vaso-vagal syndrome; (3) coved and sometime dynamic saddle-like precordial ST segment elevation resembling a right bundle branch block pattern (Figure 1). These patterns are sometime seen only in high precordial ECG recordings; (4) prolonged PR and HV intervals and left axis deviation; (5) induction of the ECG pattern with class 1c drugs. (6) Epicardial (but also endocardial) recording of a delayed conduction at the (RVOT); (7) easily inducible VF from this site; (8) un-rare association with sick sinus syndrome and atrial fibrillation; (9) minor structural abnormalities of the right ventricle at routine investigations, but significant fibrosis of the conduction system, and fibro-fatty replacement of the RVOT in rare necropsy studies. In the following thirty-six years this syndrome received more than 7000 scientific contributions in medical literature, second only to the long QT syndrome, with a lot of heavy controversies on its pathophysiology. The syndrome was originally described by Andrea Nava [1,2,3,4] as a disorder of depolarization, caused by localized structural abnormalities of the right ventricle but in the following decades a functional abnormality of depolarization was the worldwide common accepted explanation, with many elegant but inconclusive studies following this assumption. Unfortunately, there was a competition between different centers to collect a personal series and a higher number of cases rather than to carefully examinate, classify, and follow the patients according to evidence-based rules and not to emotion. This has led to an incredible collection of healthy people with PER, rather than with the true syndrome. A common international registry was initially proposed by the Brugada but never succeeded. Every arrhythmia institute preferred not to share their data claiming self-authority. A copy-and-paste habit of the last ongoing theories were rarely the rules of more than 7000 mainly inconsistent papers. The big mistake was the lack of agreement on a common gold standard for the diagnosis and investigation. Nowadays the initial theory has been re-discovered, and serious evidence-based data are little by little clarifying the pathogenesis, An incomplete advance has been made in the correct diagnosis, risk stratification, and therapy, and the same patient (or healthy subject) receives a different management in different centers. After nearly 40 years from the discovery, it may be useful and fair for new generations to know the history of this important medical advancement and of the doctors who contributed to its elucidation.

## 2. The History of the “Precordial Early Repolarization (PER)” and Sudden Death Syndrome

**Figure 2:**
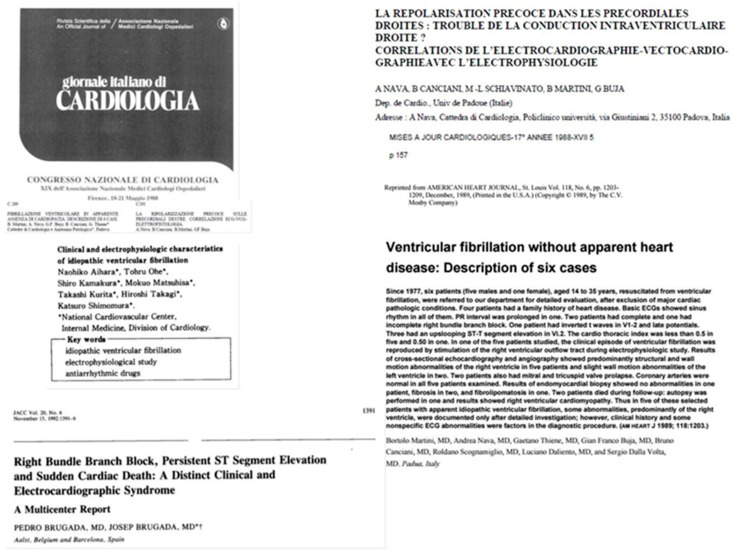
The first articles that described the syndrome [1,2].

**Figure 3:**
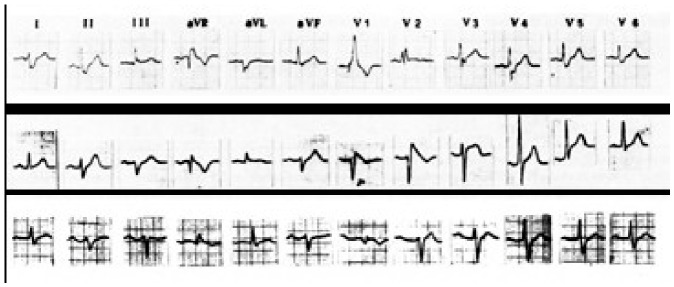
The first ECG traces of three out of six victims of aborted sudden death: Patient 1 upper line, patient 2 middle line and patient 3. Patient lower line. Patient 2 is the first and typical patient with the syndrome, but also patients 1 and 3 have possible features. See text for discussion.

On 20 October 1984 a 42 year old healthy and previous asymptomatic cook, quietly talking with the post officer outside his restaurant [9] collapsed. Fortunately, an ambulance arrived promptly, and a successful defibrillation of VF was performed by Dr. Giuseppe Piccoli at the Hospital of Conegliano [10], Italy (the land of Prosecco wine). The ECG taken showed a coved ST elevation in the precordial leads with inverted t waves in V1 and V2 and no significant s wave in V6. An acute myocardial infarction was suspected, although the initial brilliant ECG diagnosis was “focal block of the right bundle branch” [10]. Left ventricular and coronary angiography performed in Padova excluded coronary heart disease, and an electrophysiologic study was inconclusive. At that time right ventricular angiography was rarely performed because the right ventricle was not considered the source of ventricular arrhythmias, despite the pivotal study of Marcus et al. [11], and the relevant report by Thiene et al. [12]. All the ventricular arrhythmias, unrelated to evident heart disease, were retained “idiopathic”. The patient was lately studied by a 2D echocardiogram that only showed a moderate dilatation of the RVOT, and minor wall abnormalities. Additionally, this simple non-invasive technique was largely ignored until Scognamiglio enhanced the importance of this study in detecting subtle structural and wall motion abnormalities of the right ventricle, possibly consistent with minor forms of arrhythmogenic cardiomyopathy [13]. The patient was treated with amiodarone, which was later substituted with beta blockers because of thyroid abnormalities. Twenty years later, he received an implantable cardioverter/defibrillator (ICD) because of VF induced during an electrophysiological study performed by another group. The ICD has never delivered any shock, and he remains alive and well. His ECG taken regularly has shown an intermittent variation of the ST morphology (type 1 and 2), and interestingly an epsilon wave has lately appeared [14] indicating a structural and electrical evolution of this syndrome. In May 1988, a group of Cardiologists and Pathologists from Padova University described at two conferences held in Florence and in Paris [1,2,3] and in an article published in the American Heart Journal (after rejection by a major Medical Journal where the type 1 ECG was retained normal) [4], the new syndrome that included the association between a lethal clinical event called aborted sudden death due to VF (that usually starts as a polymorphic ventricular tachycardia), minor structural abnormalities of the right ventricle, and a coved ST patterns in the precordial leads (Figure 1, Figure 2 and Figure 3).

**Figure 4:**
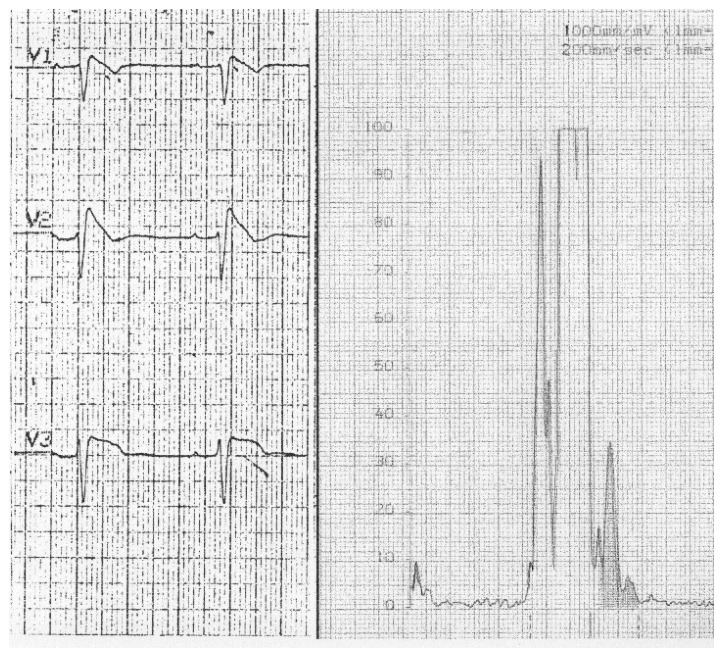
Late potentials recordings in patient 2.

The initial definition was Precordial Early Repolarization Syndrome (PERS) [3]. Of interest, the patient initially reported also had an ECG pattern of left axis deviation and slightly prolonged PR interval. An incomplete right bundle branch block (iRBBB) was excluded because of absence of a significant r1 wave in aVR and s wave in V6. Furthermore, differently from iRBBB, the vectorcardiographic loop (Figure 1) [3] showed a right-posterior-superior delay of the terminal QRS, and at endo cavitary recordings the ST elevation was coincident with a delayed depolarization of the QRS [3]. This abandoned technique has been brilliantly reevaluated by Riera, as it clearly and simply distinguishes the ECG pattern of the syndrome from typical iRBBB [15]. This conduction delay was again confirmed by the first late potential study with signal averaged ECG [16]. The presence of late potentials, (that can be found in 80% of the subjects with a type 1 ECG pattern: Figure 4) should have excluded by definition, a functional origin of the ST elevation, but was less considered [17]. Less frequently late potentials are detected with a type 2 pattern possibly indicating a true functional component of this ECG pattern.

Two more patients (number 1 and 4 of ref. [4]) had an abnormal ECG pattern, clearly not the same as patient 3, but so like the subsequent case published in the medical literature as belonging to the syndrome. Patient 1 was a 31-year-old man who had a cardiac arrest at rest. His ECG showed right axis deviation, and an RBBB with a mild coved ST elevation in V1. His ECG trace was like patient 1 and 2 of the Brugada series [6]. The angiogram of the right ventricle and the endomyocardial biopsy showed some structural abnormalities. Ventricular fibrillation was induced; he was put on amiodarone but died whilst sleeping 1 year later. Patient 4 was a 31-year-old man admitted to the emergency department in Bergamo Northern Italy, because of ventricular fibrillation that occurred while drinking coffee. His ECG showed a prolonged PR interval, left fascicular hemiblock, and again a coved ST elevation in V1. In a later ECG, he showed a more pronounced coved precordial ECG pattern [18]. He had a previous syncope 5 years before [18,19]. He was submitted to cardiac angiography and an electrophysiology study that only documented a prolonged HV interval (70 milliseconds). He did not receive any drug treatment and died suddenly at rest on 27 November 1985. His autopsy study was consistent with Lenegre disease associated with fibrofatty replacement substitution of the right ventricle myocardium [18,19,20]. The heart weighed 350 g; the left ventricle and the coronary arteries were normal. The right ventricle was enlarged (with preserved wall thickness), with marked dilatation of the pulmonary infundibulum, interstitial fibrosis, and remarkable myocardial atrophy with transmural fatty replacement of the free wall, the infundibulum, and the moderator band. Results of histology examination of the specialized atrioventricular junction showed marked fibrosis of the bifurcating bundle of His and proximal bundle branches. In these cases, the histologic structural abnormality was like Arrhythmogenic Right Ventricular Cardiomyopathy/Dysplasia (ARVC/D), but differently from this entity the central and the peripheral (at the RVOT level) conduction tissue were severely involved. The central lesion could explain the prolonged PR, HV interval, the left axis deviation, and the similarity to an iRBBB. The ST elevation could be produced by a lesion of the Purkinje network at the infundibular level, representing an unusual conduction disturbance and not a repolarization abnormality. Most of his family had asymptomatic similar ECG patterns and a mutation of SCN5A gene. One of his nephews had a cardiac arrest in 2019 during soccer when he was 14 years old. His ECG showed prolonged PR, minor left axis deviation, and incomplete right bundle branch block (RBBB) with mild ST elevation in V1. The ajmaline test was negative and there was moderate dilatation and hypokinesia of the right ventricle, without tissue abnormalities on the nuclear magnetic study. In an electrophysiology study, the HV interval was 65 ms, polymorphic VT was induced, and an ICD was implanted. His genetic study showed SCN5A and MYPN gene mutations.

## 3. The Precordial Early Repolarization (PER) ECG Pattern

The PER mainly consists of two patterns: type 1 with a coved ST, and type 2 with a saddle back morphology [21]. From time to time a same patient can show a dynamic change of the two patterns, or type 2 can be converted into type 1 by class 1 c drugs. The isolated ECG type 1 pattern of PER in the precordial leads was previously described by Osher in 1953 [22] in three healthy young men and the author explained this pattern as follows: “This is apparently due to prolongation of the depolarization process by right bundle block or possibly focal block with delayed activation of a portion of the right ventricle: unusually early onset of repolarization may also play a role”. After this paper, the PER was also described in the left lateral and inferior leads, but it is a matter of discussion if and how the two entities are related [23]. The same year, Osborn [24], published an experimental work in which a similar ECG (in lead II and not in the precordial leads), was induced during hypothermia and acidosis and was associated to VF. The ECG pattern showed a sharp hump following the QRS wave (and not fused with the QRS), in the peripheral leads, also associated to QT prolongation. Osborn forgot however to quote the paper by Tomaszewski in 1938 [25]. Interestingly this author attributed this ECG abnormality to a delayed repolarization. It was also lately demonstrated that a precordial ST elevation is temperature dependent and can be induced both at hypothermia and hyperthermia.

After Osher, Edeiken [26] published ten more healthy cases of precordial ST segment elevation, mostly with a type 2 saddle back morphology. It is of interest that in these and Osher’s cases there were not a prolonged PR interval, axis deviation and fragmented QRS, findings that were lately associated with the true syndrome. He first made a very interesting observation: this ECG pattern enhanced in the upper precordial leads. He confirmed Osher’s theory that the RS-T segment was a portion of the QRS complex with some intraventricular conduction delay. In 1975, Aldo Calo [27] published a very nice example abnormal precordial ECG with a slurred QRS and coved ST in a healthy man. He confirmed that the pattern was enhanced in the upper precordial leads and made an interesting observation: the QRS duration in aVR, L1, and V6 was normal, and s waves in these two latter leads were almost absent. His conclusion was that the ECG pattern was induced by a conduction disturbance of the upper part of the heart, namely the RVOT. Calo concluded with a wise suggestion that nowadays is so important: “The danger of cardiac iatrogenic heart disease stemming from misinterpretation of similar or even less abnormal electrocardiogram has been stressed, but unfortunately one still encounter patients who were made cardiac invalids because of one of these errors”. Nowadays little has changed and less attention is made to the psychological consequences that a clinical unproved diagnosis can create [28,29].

This assumption of a benign course of precordial ST elevation persisted until Andrea Nava clearly demonstrated by recording Endo cavitary potentials with an old Mingograph (Siemens Elema) device that the coved ST elevation is related to an endocardial delayed conduction at the RVOT level [3]. One similar Japanese paper followed the Italian observations in 1990, where one of the patients had a type 1 and another had a type 2 ECG [5]. The new syndrome, however, did not receive any attention until the paper of Pedro and Joseph Brugada, eminent experts of idiopathic arrhythmias.

In 1986, a 3-year-old Polish boy was referred to the Brugada brothers after multiple episodes of syncope [30]. His ECG showed an ST-segment elevation in leads V1 to V3. His sister displayed a similar clinical and electrocardiographic profile and died at 2 years of age. In the succeeding years, six additional cases came to their attention, and in 1991–92 they reported these eight cases as a presumed new and distinct clinical entity [6,7], named “syndrome of RBBB, persistent ST segment elevation and sudden cardiac death”. These authors previously published [31] a similar paper the same year of our detailed observation [4]. At that time, they did not publish the ECG of the patients but described two cases with iRBBB. Six patients of the new paper had the typical type 1 ECG pattern while in two the ECG (part one and two) was less typical and like patient 1 in our series [4]. It is noteworthy that in the Brugada series the ST elevation was described as a coved persistent morphology, different from later observations, and probably consistent with a more severe form. The Brugada confirmed a male predominance, a familial involvement, a prolonged HV interval, and an initiating arrhythmia that was a polymorphic ventricular tachycardia, rapidly degenerating in VF. They also firstly observed in one case an arrhythmic storm related to fever and proposed that Implantable Cardiac Defibrillator (ICD) was a better therapy compared to amiodaron, diphenylhydantoin, and beta blockers. The major difference from the Padova original paper was the presumed absence in this paper of any structural abnormality following a right and left angiographic study and an endomyocardial biopsy performed in three. They did not however give an evidence-based explanation on their finding of a prolonged HV interval, and they speculated that the pathophysiologic mechanism “was likely to be either a marked dispersion of refractoriness of cardiac tissue or extreme anisotropic properties of the conduction system and the ventricular muscle” [7].

## 4. The Initial Heavy Controversy

Following a case report by Proclemer [32] who described a case of apparently idiopathic syndrome, Naccarella [33] commented that this patient had possibly a latent ARVC/D. He also confirmed the Edeiken observation [25] that the typical ECG pattern was best recordable in the upper precordial spaces. His comment triggered an heavy reaction by Pedro Brugada [34] who wrote that his idiopathic syndrome was different from the one from Padova and accused Naccarella to “try to confuse the cardiology world”. Despite Naccarella [35] insisting on defining the entity with a priority name, the syndrome was firstly named the Brugada Syndrome (BS) in Japan by an unknown author who probably did not realize their role in the future history [36]. Some authoritative disagreement came both early and later to restore the true priority [37,38,39,40,41,42,43], but, according to Stigler’s law [44] of eponyms (this law says that no scientific discovery is named for its original discoverer), the term BS has been imposed. Fairly, Pedro and Joseph Brugada recently admitted that they were not the first describers of the entity but were only so lucky to play a major role in the history of the syndrome that received their name [45]. Despite some controversy their role was and is indeed outstanding [46]. Nowadays the syndrome has been classified as one of the J wave syndromes [47].

## 5. The Rush Years for Discovering New Features of the Syndrome

Since 1994, an increasing number of papers described different patterns of the syndrome, and some of these added new original data. D’Onofrio first publish a fatty substitution of the RVOT at magnetic resonance imaging [48]; Corrado confirmed an organic substrate and documented a familial involvement [18,20]. In the far east Sumiyoshi [49] first described a variability of the ST segment and the spontaneous change from coved to saddle-back of the ST segment, and Shimada described a case of sustained ventricular tachycardia that is very unusual in the syndrome [50], as well as the QRS morphology with a RBBB pattern described by Allocca [51]. A non-rare arrhythmia in these patients is atrial fibrillation that also may be linked to SCN5A abnormalities [51], and other supraventricular arrhythmias [52,53,54]. Atrial fibrillation can be associated with a high vagal tone and a low sympathetic tone that might be discussed properties of symptomatic BS [55].

The first epidemiological study arrived again from the far east and France, where Tohyou [56] and Hermida [57] reported a 0.05–0.1% prevalence of the coved pattern, and a 6% of some saddleback deformity in an apparently middle aged healthy population from Japan and north-western France. The French analysis probably still represents the true distribution of the ST pattern. Subsequent studies and meta-analysis [58] presented more severe data, but these series included people with purely iRBBB, fever, drug induction, and high intercostal recordings that have create an unjustified pandemic [59]. If we establish that all the above asymptomatic subjects might have an underlying syndrome and not a benign ECG pattern it is not difficult to exceed 5% of the world population.

The prevalence of the true syndrome is indeed one of the main clinical concerns that still lacks scientific evidence because of confusion between syndrome and ECG [60]. The initial report [61] ranging between five and sixty six per ten thousand indicated that with a world population of 6,157,000,000, that would mean between 3,000,000 and 40,000,000 have BS, similar in scope to the estimated 36,100,000 infected with HIV [62]. A recent multicentre study [63] collected 678 true patients in the entire world. These patients that come from the most prestigious centres surely underestimate the problem but do not justify the increasing media reports that ascribe to BS all the cases of juvenile sudden cardiac death. In our limited experience, we have seen two patients with cardiac arrhythmias and with the true syndrome in the last 25 years in a population of 190,000 inhabitants [64,65]. This is similar to a study conducted in Denmark, where serious epidemiological data are available [66].

In 1996, Electrophysiologic testing initiated to be a mayor diagnostic technique for risk stratification [67], but it resulted in a poor 60% sensitivity. Later, different protocols enhanced the sensitivity, but not the specificity [68,69,70].

A relevant observation came from Thailand where Nademanee related the ECG pattern [71] to the high incidence of sudden death among young Southeast Asians males who died unexpectedly during sleep: the sudden unexplained death syndrome (SUDS).

Another important discovery came from Miyazaki [72] who evaluated the multiple effects of autonomic and antiarrhythmic drug modulation and concluded that the ST elevation could be enhanced by alpha-adrenoceptors or muscarinic stimulation or class 1A drugs, whilst it was mitigated by beta adrenergic stimulation or an alpha adrenoceptor blockade. At present it should be ethically difficult to repeat such a human experiment that indeed demonstrated that these patients unusually resist to aggressive multiple drug treatment, differently from all the therapeutic cautions currently applied. This author noticed that class 1c drugs augmented ST elevation, and Brugada brilliantly proposed a drug challenge, initially with ajmaline, and lately with flecainide (being the first more sensitive), and procainamide to induce the type 1 ECG in otherwise normal or mildly abnormal ECG [73,74], leading to an epidemic of presumed patients affected by the syndrome [75]. Nowadays the problem is far from be resolved as so many tests are performed to asymptomatic people or to people with low probability to have the syndrome [76]. It has been demonstrated that the ECG pattern can be induced in 3% of healthy volunteers with a normal ECG [77], and more frequently in asymptomatic people with minor precordial ST abnormalities. In the absence of worldwide accepted evidence-based data and serious guidelines this incorrect medical behaviour has severe health and legal implications both for those who abuse the test and also for those who do not [76]. It is not irrelevant that despite their diffuse availability and relatively safe profile, some major ventricular arrhythmias and electromechanical dissociations have been described as complications [78,79]. The drug challenge has also been also introduced to enhance the areas of epicardial depolarization abnormalities prior to ablation [80], but this procedure has not received a consensus for asymptomatic people [81]. The drug-induced coved type pattern has shown a good prognosis, as Shimizu calculated a yearly risk of lethal events of 0.2% [82]. In another analysis performed by Viskin, the risk of a spontaneous VF on asymptomatic patients was only 0.3% per year [75]. A low rate of arrhythmic events was also reported by many others [83,84,85]. Ajmaline is a drug that has been extensively used throughout the electrophysiological world. Its main role is to enhance various degrees of conduction defects. What we can derive from evidence-based studies, is that a positive ajmaline test, nowadays, does not provide any clear additional information on the risk stratification for major ventricular arrhythmic events on asymptomatic individuals with a non-diagnostic Brugada ECG pattern. This conclusion reaffirms the wise statements of Sami Viskin: “To conclude, a note of caution: in a recent study simulating screening for Brugada syndrome, as many as 45% of healthy control subjects had minor imperfections in the right precordial leads that could be interpreted as type 2/3 Brugada ECG. We are forced to wonder how often asymptomatic individuals enter a path to rule out BS for the wrong reasons, have a false positive ajmaline test followed by a positive EP study, and faced with the alternative of facing sudden death, ends up with an unjustified ICD implantation: A positive ajmaline test does not always mean you have BS” [75].

The induction of type 1 ECG in healthy subjects was not limited to class 1 c drugs, but antipsychotic and other different drugs have increasingly been described with a similar effect [86], usually at toxic levels. Postema [87] has published a world-wide use recommendation on drugs that may induce the ECG pattern and for this reason should be strictly forbidden [88]. This paper was followed by a more extensive series [89], but both papers have however many limitations regarding the true prevalence of the clinical observations that fortunately are so rare and anecdotal. In this paper [88], Konigstein examined web data and found 74 cases of noncardiac drugs that induced the ECG pattern. “Among these cases, 36 (49%) were induced by psychotropic drugs, 20 (27%) were induced by analgesic–anaesthetic drugs, and 18 (24%) were induced by other noncardiac drugs. The most frequently reported agent was intravenous propofol, which accounted for 20% of cases. The most frequently reported oral agents were lithium and amitriptyline (accounting for 20% and 16% of cases caused by oral drugs, respectively). Mostly had the ECG pattern during toxic doses, and 13,5% died. It is not however yet well demonstrating the true arrhythmic risk of class 1 c drugs, amiodaron, and beta blockers to induce major events apart from the ECG appearance of pattern 1 or 2. Additionally, Postema fairly wrote that “Our study does not provide information about the incidence of drug induced Brugada syndrome. Publication bias may have resulted in significant overestimation of the mortality”. These wise considerations can also be referred to different cardiac drugs inducing the ECG pattern, but not the syndrome at least at nontoxic levels. In our experiences these patterns can be induced in up to 5% of those who utilize those drugs, and that usually do not have any event at follow up. “Furthermore, whether ECG screening or brief hospitalization during the initiation of therapy will be of value in preventing drug-induced Brugada syndrome is unknown”. The pivotal pharmacological studies of Miyazaki relied on a supportive “in vitro” laboratory demonstration by Antzelevich [90,91] who explained “*using a newly developed arterially perfused canine ventricular wedge preparation, we provide, for the first time, direct evidence in support of the hypothesis that heterogeneous distribution of an Ito-mediated spike-and-dome morphology of the action potential across the ventricular wall underlies the manifestation of the electrocardiographic J wave*”. The presence of a prominent action potential notch in the epicardium but not in the endocardium is shown to provide a voltage gradient that manifests as a J (Osborn) wave or elevated J-point at the R-ST junction of the ECG. This theory, never demonstrated clinically, was believed to be the Holy Grail for all who supported a functional origin of type 1 ECG.

The syndrome has indeed some genetic traits with an autosomal dominant pattern. After the paper by Brugada [7] and Corrado [20] who described familial traits in patients with the syndrome, a major advance in this assumption came from the paper by Chen [92] who described some genetic abnormalities of the Sodium channels SCN5A underlying some of the subjects with the ECG. The abnormality was largely diffused in the patients evaluated in their study, but its high prevalence was not confirmed recently by the same group [3]. Nowadays SCNA abnormalities are found in 20% of people with PERS [93,94], but are not specific for the syndrome [95]. The prevalence is higher in patients with the true syndrome [96,97,98] and more extensive epicardial abnormalities, but rarely some relevant discrepancies between phenotype and genotype have been reported [99]. A single case of a family with homozygous traits has been also described [64]. Despite relevant studies a familial history and a positive genetic trait have no role in risk stratification for sudden death, at least as an isolated risk factor.

**Figure 5:**
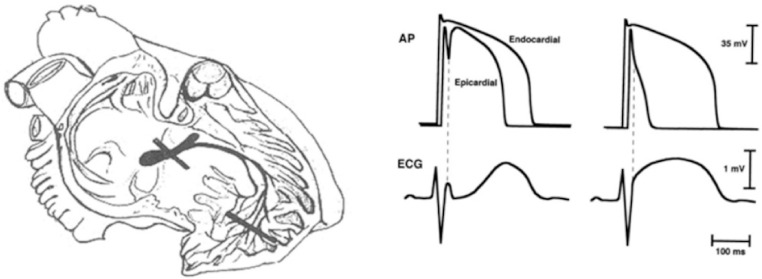
Schematic drawing of the two theories. On the left the organic theories that ascribes the ECG pattern to a double lesion of the conduction tissue: the main bundles and the Purkinje network of the RVOT (Drawing by Sergio Cannas). On the right the functional theory that ascribed the ST changes and consequent arrhythmias to different action potentials at the epicardium and at the endocardium.

After this initial series of patients, the Brugada started to collect similar cases of true syndrome from other centres, rapidly reaching in six years to 63 cases with type 1 ECG, mostly symptomatic or at high risk [100], with 80% of inducibility at electrophysiologic study (EPS), with poor response to medical therapy, and with high rate of major event at follow up both for symptomatic and asymptomatic patients. A couple of years before, Atarashi [101] published the first Japanese series, mainly with limited clinical data, but indeed published a beautiful example of dynamic ECG pattern in the same patient. Alings and Wilde [102] presented in 1999 the third relevant series, and suggested that 40–60% of the patients affected by idiopathic VF had this entity. Differently from our hypothesis they favored at that time the functional theory “*Both theoretical considerations and* in vivo *experiments support the idea that heterogeneity of repolarization across the wall of the RV outflow tract (RVOT) contribute to the ECG patterns and the genesis of arrhythmias in the Brugada syndrome*”. Their drawing of this hypothesis (Figure 5) was so different from ours but had a relevant impact in the following two decades. Dr Wilde has later fairly accepted and demonstrated other possibilities [42].

These initial series of true patients and asymptomatic people with the PER pattern stimulates other centres to make their own collection, often including some subjects of other series. This run has led to reports of 1200 individuals almost all asymptomatic and often with a less typical or only drug-induced ECG [103]. Unfortunately, the only conclusion that can be drawn from these big series is that the syndrome is so rare, but the ECG is not, especially if drug induced.

A relevant observation came from Belhassen and Viskin [104] who provided evidence that quinidine, that they had used successfully for idiopathic VF in the last two decades, was also effective for the syndrome.

Martini et al. and Gussak et al. [105,106], closed the last decade of the twentieth century with two very extensive and accurate reviews. The first author emphasized the concealed organic substrate underlying the syndrome, and its depolarizing origin. Gussak (the unknown discoverer of short QT interval syndrome), insisted that “The available data suggest that the Brugada syndrome is a familial primary electrical disease caused by a defect in an ion channel gene, resulting in premature repolarization of some right ventricular epicardial sites”. Gussak however fairly but briefly admitted that a concealed structural heart disease could not be excluded. He also presented a list of different conditions that could induce a Type 1 pattern: the so called phenocopies, well described in the following decades by Baranchuk [107].

## 6. The New Century

Gussak [108] initiated the century with a new interesting topic: the similarities between the rare PERS and the frequent early repolarization of the inferior and lateral leads [23], that lately also became a syndrome (ERS) after the paper from Haissaguerre [109]. The author discussed the cellular bases of the two syndromes and the significance of the J wave, insisting on the role of ion channels and of different transmembrane action potentials in epicardium and endocardium but fairly admitting “*that all of these proposed mechanisms remain to be shown in humans and that these hypotheses remain to be rigorously tested*”. Later, these authorities confirmed the priority of depolarization also at the origin of the J wave syndrome [110]. Nowadays both BS and ERS are classified as J wave syndromes.

It was in 2002 [61] that a group of experts proposed some guidelines for the syndrome. They divided the ECG pattern of the syndrome into three types. A severe bias was introduced because they considered only the first three precordial leads, without any detail for the remaining nine leads and for A-V conduction. After a “decade of copy and paste” articles, very few scientists remember that this classification was only based on a single case report seen by Dr. Corrado, that illustrated the dynamic precordial ECG-leads of a resuscitated patient [61]. This unreliable classification has been unfairly abused, and asymptomatic healthy individuals incidentally found (both spontaneously or drug related) to have one of these three patterns have been submitted to invasive studies and therapies. Corrado proposed later a simple method to distinguish athlete’s heart [111], and a group of ECG experts have proposed a new classification that again mostly is based on indexes, angles, etc., rather than on clinical features [112] Following the limitations derived by the first classification regarding confusing differences between type 2 and 3, this expert group has presented new corrective criteria. Only type 1 and 2 are considered and the differences between the typical ECG and different conditions, like incomplete RBBB, ARVD, pectus excavatum, rSr1 pattern [91,106,107], and others are highlighted, with re-evaluation of an old technique: vectorcardiography [3,15,113,114,115]. The Prelude study [116] has later stressed the important detection of fractionated QRS (fQRS), indicating a depolarization disorder that confirms some similarities with the epsilon waves [11,14]. Calò [117] videnced the importance of s wave in L1 as a marker of risk, and Migliore [118] reaffirmed the significance of associated atrio-ventricular conduction disturbances. Not only VF but also other severe conduction disturbances may be the cause of syncope in many of these patients [119]. The conduction disturbances are more frequent when SCN5A abnormalities are present and are significantly associated with major cardiac events [120]. In inexpert hands, the ECG has unfortunately become a lethal weapon like in Calò’s years [27], because some similar ECG patterns are not so rare, and it is so difficult for an inexperienced doctor not to tell the healthy subject that he may have BS following an inconclusive ECG recording. The ECG pattern has been documented in more than one hundred different unrelated situations, and this unfortunately diminishes the specificity of this finding [121]. It has been frequently repeated that an ECG is not a syndrome [59,122], but this assumption is so rarely considered, and the impulsive strategy is the indiscriminate collection of hundreds of presumed cases, only relying on minimal ECG abnormalities, drug challenge, or lead position. These high precordial leads recordings [31], in cases where it is not recordable in the usual fourth intercostal space, have gained much more importance than he merits. This topic followed body surface mapping studies by Shimizu [123], who showed that maximum ST elevation was distributed in an area of the right ventricular outflow tract in all type 1 patients. The maximum elevation was located on the second intercostal space in the patients in whom only a mild saddleback-type ST elevation was seen in leads V1 and V2 of the 12-lead ECG. In patients with SUDS, Sangwataroi demonstrates the usefulness of high intercostal recording, especially if recorded during drug challenge [124]. Hisamatsu [125] demonstrated that when the ECG was recorded in the third intercostal space, a Brugada-type ECG (mostly type 2), was found in 12 (5.8%) of 206 subjects, with minimal abnormalities in the precordial leads. The same data were not confirmed by Shi who found only 1.5% prevalence of type 2 pattern [126] and Holst [127] who reported a 4.7% non-specific prevalence of type 2 and 9.4% of type 3, by recording at different intercostal spaces among 340 healthy European individuals. These data were also confirmed in Turkey [128], and in more than 10% in Chung series (type 2 and three) [129]. Later Govindan [130] demonstrated that a predominant high precordial type 1 pattern can easily induced following ajmaline in 42% of those that have a positive drug test (17% in his selected population suspected to have the latent syndrome). These authors correctly expressed concern over their prognostic value and specificity of these findings and in the new guidelines [131] the high precordial recordings of the type 1 pattern is no longer considered diagnostic of the syndrome like in the previous ones [132].

Another relevant situation related to the syndrome was the occurrence of sudden death during fever initially reported in one patient [7], well re-described by Gonzalez Rebollo [133] confirming previous laboratory reports [134]. The type 1 pattern usually disappeared in the afebrile state and was not associated with lethal events [135]. Some dangerous reports of this association came however from Junttila [136] with very few details, while Adler in an epidemiological survey [137] again did not see any lethal case. In his paper, a fever >38 degrees induced type 1 pattern in up to 3% of the middle-aged population, and another 2% showed type 2. In the afebrile state this pattern mostly disappears and is rarely inducible with drug challenge. Thus asymptomatic people with an only fever inducible type 1 ECG were considered at so low future risk of arrhythmic events, but this was not confirmed in a recent meta-analysis [138]. In another remarkable series [139] that probably included the previous cases of the same group, fever counted for 6% of 588 patients with the syndrome and cardiac arrest. Children and patients with SCN5A abnormalities were at higher risk [97]. Noteworthy, 71% of these patients had an abnormal spontaneous ECG outside fever, and also in a less clear series of Amin and Tsai, similar data were presented [140]. Zagidullin has observed that some J wave was present only in the acute phase of COVID-19 [141], which could account for enhanced arrhythmogenesis during fever. The recent guidelines [130] however do not carefully indagate the difference between type 1 onset only during fever, and preexisting type 1 accentuated by fever, and propose a class 1c for all people with type 1 ECG induced by fever. The cited references do not support this statement. The topic is of extreme interest as there is a very high risk to overestimate the incidence of cardiac arrest during fever, only relying on rare case reports. No evidence-based data at present time are available on the prevalence of the PER in all population with cardiac arrest and fever.

## 7. The Great Debate: Functional or Organic Syndrome?

The premature classification of the syndrome among the channelopathies [142] has not received univocal consensus and will be probably ameliorated in the future [142,143]. Nowadays all the authors that have initially advanced a functional theory agree that a structural abnormality of the RVOT underlies a depolarization abnormality and the spontaneous or inducible ECG pattern [42,45,143]. This is a fair admission from outstanding scientists of the pivotal theories of a great humble scientist, Andrea Nava [8], and of the school of Medicine in Padova [10,21,41,144,145,146]. The path toward the acknowledgement of the organic substrate of this new syndrome was full of difficulties despite that a normal heart belonging to a patient with the syndrome has never been reported, in a detailed necropsy study, both in the far east and in western countries [10]. It is difficult to understand why only anecdotal case reports and not systematic studies have not been performed in people who died suddenly with this suspected pathology. Likely the initial indisputable assumption of a functional entity created a wall against different opinions and evidence. Anecdotal studies by Gotoh and Kirshner [147,148] evidenced significant histological lesions of the conduction system, but not of the myocardium, in the vast majority of Asians who died suddenly at night (in whom the syndrome could retrospectively been suspected also if no ECG was available). Fibrotic substitution and some fatty changes were noted both at the sinus node level and at the His and bundles level. After the first description of an underlying pathology underlying the syndrome [4], at least 18 more reports confirmed this finding [21], but were totally ignored until the paper by Coronel [149], who provided evidence of fibrotic changes of the RVOT. Unfortunately, the conduction system was not examined in detail and Coronel concluded that “In this patient with BS, conduction slowing based on interstitial fibrosis, but not transmural repolarization differences, caused the ECG signs and was the origin of ventricular fibrillation”. It is of historical interest that some years before, the Brugada brothers submitted to Guy Fontaine [150] an autoptic case of presumed idiopathic BS, that on his opinion was a typical case of right ventricular cardiomyopathy, not agreeing with a less expert pathologist who had made a diagnosis of normal heart. This case, that (for what we know) was never published in detail, illustrates the complexity of autoptic examinations in the cases of sudden death, needing to be performed by an expert team. Most of the autoptic cases had some structural RV pathology mainly at the RVOT, but also a sclerotic–degenerative lesion of the His bundle, of the RBB, and of the RVOT, similar to what was described by Jean Lenegre in 1963 [151]. It is of interest that Lenegre syndrome shares not only a similar histologic damage but also the same SCN5A abnormalities in some cases. Of interest, in those patients with BS and SCN5NA abnormalities the conduction disturbances are more advanced [152,153]. All these patterns allocate BS closer to Lenegre disease rather than to ARVD/C. In addition to the central lesion that explains the prolonged HV interval, the prolonged PR, and the axis deviation, the typical pattern of the syndrome is the histologic lesion (mainly epicardial) that induces a conduction disturbance at the RVOT level and the abnormal elevated ST segment elevation. Apart from the fibrotic lesions of the RVOT, the peripheral Purkinje system of the RVOT might be also affected and Haissaguerre succeeded to cure the initiating ventricular arrhythmia with ablation of abnormal Purkinje activity and modification of the ECG pattern at the inner RVOT [154,155]. Since this paper has many interesting contributions, the electroanatomic mapping of this delayed activity has been introduced [156], and the ST elevation has shown to be normalized both by endocardial and epicardial ablation [80,154,157]. The underlying pathophysiology is however still well concealed within the RVOT epi-myocardial layers and in the peripheral Purkinje system [143,157,158,159]. In the year 2007 after the organic and the functional theory, Elizari entered the run by proposing the theory of an abnormal expression of cardiac neural crest cells migrating to the RVOT and was responsible for the structural abnormalities [160,161]. In this area, Durrer demonstrated a long time ago areas of delayed QRS [162]. The vast majority of RVOT ventricular tachycardias [163] and ventricular extrasystoles (both septal and free wall) arise from myocardium within the first 1 to 2 cm beneath the pulmonary valve. Despite similar morphology, the pathophysiology of these arrhythmias is different. The occurrence of any form of ventricular arrhythmia arising from the RVOT brings to mind its probable relationship with the same or an analogous substrate of Brugada syndrome [163] and a similar and peculiar embryogenesis and development of this zone of the heart. Boukens [164] stressed that the heart is formed from several progenitor regions: the first heart field predominantly forms the left ventricle, whereas the second heart field forms the right ventricle and outflow tract. Furthermore, the embryonic outflow tract consists of slowly conducting tissue until it is incorporated into the ventricles and develops rapidly conducting properties. The last hypothesis on PERS pathophysiology has come from Corrado [165]: “*Whether ARVC and BS actually have a common pathogenetic denominator remained unsolved by past clinical and pathologic studies. However, recent experimental studies have renewed the interest in identifying the mechanisms responsible of the overlapping disease phenotype by demonstrating a subcellular interrelationship due to a crosstalk between desmosomal and sodium channel proteins*”.

## 8. Therapy and Risk Stratification

The initial drug treatment for the patients with the syndrome and cardiac arrest-included beta blockers, amiodarone, and diphenylhydantoin and demands pace-maker with poor results [4,7], excluding the first patient [9]. Soon after, implantable defibrillators became the first line therapy, followed by endo-epicardial ablation [157], which is not a worldwide recognized therapy, nor is it for asymptomatic people [80]. For low-risk people, quinidine is a favourable treatment with its use limited by a high rate of side effects [166,167]. A genetic cure might be on the road,. Risk stratification for asymptomatic people is the major problem of this syndrome and the debate is unresolved [168,169,170]. The main problem, which will be not discussed in this paper, is that there is not a gold standard for the correct diagnosis of PER/BS and that so many uncontrolled series of mostly asymptomatic people have been classified as BS. A long distance has been travelled in these last four decades, but a malignant habit to impose a self-referenced diagnosis and treatment for some still unknown entity requires, from a clinical point of view, a word of caution [35].

## 9. Conclusions

The diseases are not created and patented by contemporary physicians, as they affect the human beings since their appearance on earth. Necropsy and other sophisticated investigation studies of Egyptian mummies have detected most of the diseases that affect people of this new millennium. Doctors listen to symptoms, look for clinical signs, perform sophisticated tests, and search for the pathophysiology cure of the disease. The distinction between symptoms, signs, syndrome, and disease, despite the old teaching by Avicenna (Ibn Sina, 980-1037), in “The Canon of Medicine, are not yet totally clear and so often a syndrome or a disease is identified with a sign like a diagnosis of Parkinson’s syndrome or disease in somebody who has only trembling or a diagnosis of BS in somebody who has only a peculiar ECG. Every year new syndromes and diseases must be classified. This rarely means that they are a new entity (like COVID II infection) but only that these entities were not recognized before. Usually, the discover follows step by step observations made in different periods by different scientists. Diabetes always existed but its pathophysiology was only recognized when the Venetian doctor Vittorio Trincavella, in the fifteenth century tasted the sweet urine of a patient with polyuria. Not rarely some discoveries were identified years or centuries before but were simply forgotten because they were written in local journals, and consequent limited access to the international official literature. Not rarely different scientific interests both of authors and of the referees play a role in burying new discoveries. The syndromes and the diseases are often named with eponyms that should fairly honour the doctor who first clarified the new entity, but rarely the discovery receives the name of another author or is generically classified [171]. This well reflects the Stigler’s law of eponyms: *no scientific discovery is named after its original discoverer* [44]. When a syndrome is recognized, the history does not end but starts, as the final objective is to identify the disease, which this is essential to find the cure. If the scientific rules established centuries ago by Galileo are followed, a correct resolution of the problem can quickly be expected, but if the rules of evidence-based medicine are postponed to self-claimed scientific authority there is a high risk to lose time and research funding. The role of the referees is crucial to supervise the correct scientific pathway, but it is almost impossible that personal expectations, friendship, or antagonism do not intervene in the review process. The widespread availability of scientific media on the Internet has made it easy to identify the history of a discovery and of the steps that produced the accepted evidence of new findings. The knowledge of the history of the medical discoveries is not merely the claim for any personal expectation, but the analysis of the pathways covered by different researchers, that starting from trivial signs and symptoms led to the original discovery of a new disease. There is not progress without knowledge and respect of the past, and Doctor Andrea Nava deserves this honour [8].

## Data Availability

This is an historical review that describe the ongoing progress in the knowledge of the syndrome. No new data were created but in the reference section reported results can be found, including links to publicly archived datasets analysed or generated during the study.
